# Manipulation of Intraday Durations of Blue- and Red-Light Irradiation to Improve Cos Lettuce Growth

**DOI:** 10.3389/fpls.2021.778205

**Published:** 2021-11-26

**Authors:** Tomohiro Jishi, Ryo Matsuda, Kazuhiro Fujiwara

**Affiliations:** ^1^Department of Biological and Environmental Engineering, Graduate School of Agricultural and Life Sciences, The University of Tokyo, Tokyo, Japan; ^2^Grid Innovation Research Laboratory, ENIC Division, Central Research Institute of Electric Power Industry, Abiko, Japan

**Keywords:** artificial light, leaf elongation, leaf morphology, photomorphogenesis, light receptors

## Abstract

The morphology of plants growing under combined blue- and red-light irradiation is affected by the presence or absence of time slots of blue- and red-light mono-irradiation. The purposes of this study were to investigate the morphology and growth of cos lettuce grown under light irradiation combining several durations of blue and red light simultaneously and independent mono-irradiations of blue and red light during the day, and to clarify the effects of the durations of blue-light mono-irradiation and blue-light irradiation. Young cos lettuce seedlings were grown under 24-h blue-light irradiation with a photosynthetic photon flux density (PPFD) of 110μmol m^−2^ s^−1^ (B+0R) or under 24-h blue-light irradiation with a PPFD of 100μmol m^−2^ s^−1^ supplemented with 8 (B+8R), 16 (B+16R), and 24-h (B+24R) red-light irradiation with PPFDs of 30, 15, and 10μmol m^−2^ s^−1^, respectively (Experiment 1). The daily light integral was 9.50mol m^−2^ in all treatments. In Experiment 1, leaf elongation was promoted as the duration of red-light irradiation decreased and the duration of blue-light mono-irradiation increased. The maximum shoot dry weight was observed under the B+8R treatment. Growth was likely promoted by the expansion of the light-receptive area caused by moderate leaf elongation without tilting. In Experiment 2, young cos lettuce seedlings were grown as for Experiment 1, but blue- and red-light irradiation intensities were reversed (R+0B, R+8B, R+16B, and R+24B). Leaf elongation was promoted by the absence of blue-light irradiation (R+0B). The leaf surface was increasingly flattened, and the shoot dry weight was enhanced, as the duration of blue-light irradiation increased. Thus, cos lettuce leaf morphology may be manipulated by adjusting each duration of blue-light mono-irradiation, red-light mono-irradiation, and blue- and red-light simultaneous irradiation, which can, in turn, promote cos lettuce growth.

## Introduction

The effects of lighting patterns on plant growth have been studied to improve plant cultivation with artificial light. A combination of blue and red light can prevent spindly growth (Hoenecke et al., 1992; Yorio et al., 2001), epinasty (Seif et al., 2021), and achieve a high photosynthetic rate and high growth rate (Yorio et al., 1998; Hogewoning et al., 2010) and improve disease and nutritional status assessment (Moosavi-Nezhad et al., 2021). Consequently, lighting patterns using blue and red light have been well-studied. When plants are irradiated with blue and red light simultaneously, growth rates are highest at a blue/red photosynthetic photon flux density (PPFD) ratio of 80–90/20–10 (e.g., Hernández and Kubota, 2016) at an identical total PPFD. In these studies, the spectral photon flux density distribution (SPFD) was constant during the light period. However, recently, there have been attempts to promote plant growth using lighting patterns by applying different SPFDs during different hours of the day (Jishi et al., 2016; Ohtake et al., 2018).

Jishi et al. (2021) grew cos lettuce under various blue- and red-light combinations. Lighting patterns that include 12h of blue-light mono-irradiation (i.e., irradiation with only blue light without other colors of light) promote leaf elongation, probably owing to the phytochrome reaction. The phytochrome photostationary state (PSS) is the ratio of active phytochrome to total phytochrome; a low PSS promotes leaf and stem elongation through the shade-avoidance response (Smith and Whitelam, 1997; Kong et al., 2020). The PSS under blue-light mono-irradiation is significantly lower than that under blue- and red-light simultaneous irradiation; therefore, blue-light mono-irradiation is considered to promote leaf elongation. Jishi et al. (2021) also reported that moderate leaf elongation caused by 12h of blue-light mono-irradiation promotes growth, probably because of the expansion of the light-receptive area and the greater amount of light received, although excessive leaf elongation causes the plants to collapse and does not promote growth.

The degree of leaf elongation may be controlled by adjusting the duration of blue-light mono-irradiation (Jishi et al., 2016). The effects of blue-light mono-irradiation on plant morphology are mediated by the phytochrome reaction, and blue-light irradiation (i.e., irradiation with blue light, with or without other colors of light irradiation) affects plant morphology through the blue-light receptors of phototropin and cryptochrome (Lin, 2002). Plant morphology may also be controlled by adjusting the blue-light irradiation duration.

In the present study, we conducted a pair of cos lettuce cultivation experiments in which blue- and red-light irradiation were combined. Experiment 1 comprised four treatments with identical durations of blue-light irradiation and different durations of blue-light mono-irradiation to investigate the effects of blue-light mono-irradiation duration and possible role of the phytochrome-mediated reaction. Experiment 2 comprised four treatments with identical durations of blue-light mono-irradiation and different durations of blue-light irradiation to investigate the effects of blue-light irradiation duration and possible role of the blue-light-receptor-mediated reaction. In addition, we discuss the blue- and red-light irradiation methods that effectively promote cos lettuce growth.

## Materials and Methods

### Lighting Patterns

The PPFD values described here were measured at 2cm above the surface of the urethane cube, in which the cos lettuce seedlings were planted, using a light quantum sensor (LI-190SA; LI-COR, Lincoln, NE, United States).

**Figure 1:**
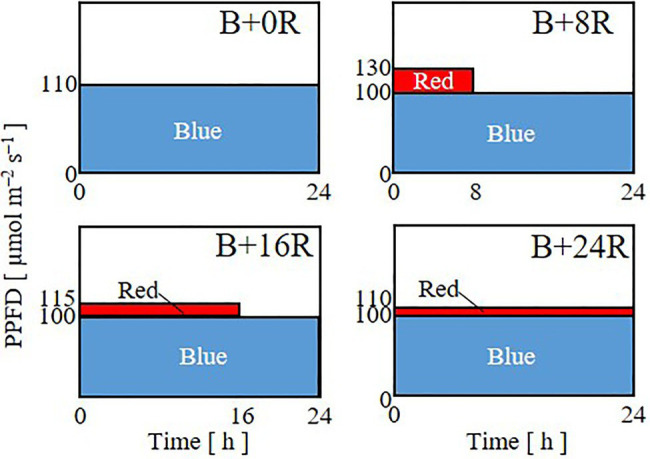
Lighting patterns used in Experiment 1. Plants were subjected to 24-h blue-light irradiation with a PPFD of 110μmol m^−2^ s^−1^ (B+0R) or under 8 (B+8R), 16 (B+16R), or 24-h (B+24R) red-light irradiation with PPFDs of 30, 15, and 10μmol m^−2^ s^−1^, respectively, supplemented with 24-h blue-light irradiation with a PPFD of 100μmol m^−2^ s^−1^.

In Experiment 1, to investigate the effects of the duration of blue-light mono-irradiation, seedlings were grown under 24-h blue light with a PPFD of 110μmol m^−2^ s^−1^ (B+0R) or under 24-h blue-light irradiation with a PPFD of 100μmol m^−2^ s^−1^ supplemented with 8 (B+8R), 16 (B+16R), or 24-h (B+24R) red-light irradiation with PPFDs of 30, 15, and 10μmol m^−2^ s^−1^, respectively (Figure 1). The durations of blue-light mono-irradiation were 24 (B+0R), 16 (B+8R), 8 (B+16R), and 0-h (B+24R). The daily averaged PPFD was 110μmol m^−2^ s^−1^ [daily light integral (DLI): 9.50mol m^−2^] for all treatments. The daily averaged PPFDs of blue and red light were 100 and 10μmol m^−2^ s^−1^, respectively, for B+8R, B+16R, and B+24R, which included red-light irradiation.

**Figure 2:**
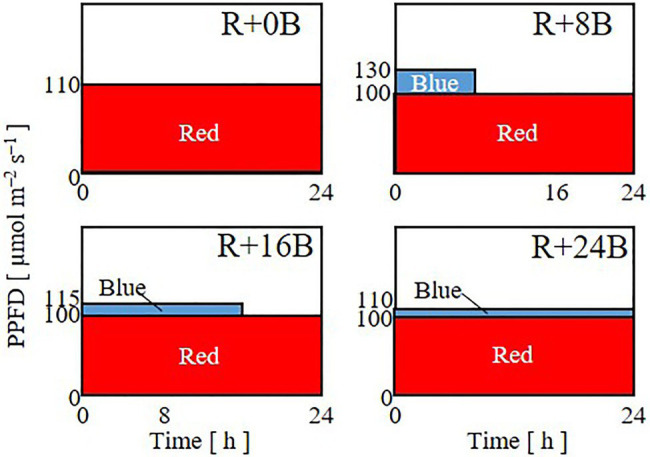
Lighting patterns used in Experiment 2. Plants were subjected to 24-h red-light irradiation with a PPFD of 110μmol m^−2^ s^−1^ (R+0B) or under 8 (R+8B), 16 (R+16B), or 24-h (R+24B) blue-light irradiation with PPFDs of 30, 15, and 10μmol m^−2^ s^−1^, respectively, supplemented with 24-h red-light irradiation with a PPFD of 100μmol m^−2^ s^−1^.

In Experiment 2, to investigate the effects of the duration of blue-light irradiation, seedlings were grown under 24-h red-light irradiation with a PPFD of 110μmol m^−2^ s^−1^ (R+0B) or under 24-h red-light irradiation with a PPFD of 100μmol m^−2^ s^−1^ supplemented with 8 (R+8B), 16 (R+16B), or 24-h (R+24B) blue-light irradiation with PPFDs of 30, 15, and 10μmol m^−2^ s^−1^, respectively (Figure 2). The daily averaged PPFD was 110μmol m^−2^ s^−1^ for all treatments. The daily averaged PPFDs of blue and red light were 10 and 100μmol m^−2^ s^−1^, respectively, for R+8B, R+16B, and R+24B, which included blue-light irradiation.

### Plant Material

Cos lettuce (*Lactuca sativa* L. “Cos lettuce”; Takii Seed Co., Ltd., Kyoto, Japan) seeds were sown on watered urethane cubes and germinated in a temperature-controlled chamber (MIR-553; SANYO Electric Co., Ltd., Osaka, Japan) at 25±1°C under a 16-h light/8-h dark cycle. Light was provided with white LEDs at a PPFD of 100μmol m^−2^ s^−1^. Seven days after sowing, seedlings with approximately 1-cm-long first true leaves were selected for the cultivation experiments.

### Cultivation Experiments

Sixteen cos lettuce seedlings were transplanted individually into four holes made in four plastic boards of each urethane cube. Each of the four plastic boards was placed on a 5-L plastic container filled with a continuously aerated nutrient solution (half-strength Otsuka-A nutrient solution; OAT Agrio Co., Ltd., Tokyo, Japan) with electrical conductivity of 0.13±0.01S m^−1^. The internal spaces of two temperature-controlled chambers were partitioned into upper and lower compartments with cardboard and black paper to prevent light contamination between compartments. Four seedlings were cultivated in each of the four compartments for each treatment. The temperature was maintained at 25±1°C throughout the day in all compartments. The CO_2_ concentration and relative humidity were not measured or controlled but were expected to have been similar among all compartments because the external air was continuously introduced into each compartment using air pumps from the same space. At 14days after transplanting (21days after sowing), all seedlings were harvested and measured. The total leaf area per plant was measured using an area meter (AAM-9; Hayashi Denko Co., Ltd., Tokyo, Japan). The shoots were dried for 1h at 100°C and then dried at 80°C for 3days before measurement of the shoot dry weight. The length and width of the largest leaf on each seedling were measured using a ruler. The cultivation experiments were repeated twice for each of the eight treatments, and the compartments were changed for each replication.

### Lighting Sources

**Figure 3:**
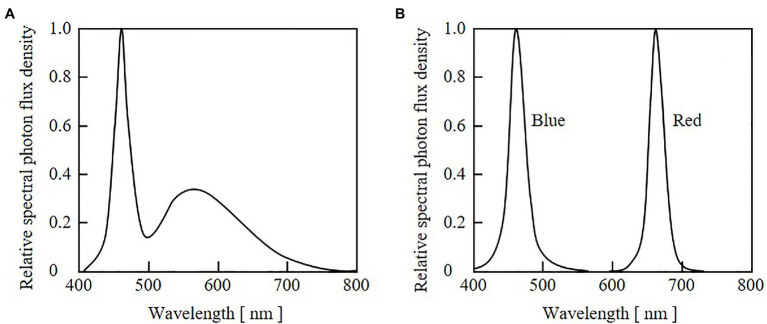
Relative spectral photon flux density distributions of light from white LEDs used for seedling cultivation **(A)** and from blue and red LEDs used for growth experiments **(B)**.

An LED panel with indicator-type white LEDs (NSPW310DS-b2W; Nichia Corp., Tokushima, Japan) was used for seedling growth before the cultivation experiments. Panels with blue (HBL3-3S55-LE; Toricon, Shimane, Japan) and red (SRK1-3A80-LE; Toricon) LEDs were used for the cultivation experiments. The relative SPFDs of the white, blue, and red LED lights are shown in Figure 3. The SPFD was measured with a spectroradiometer (MS-720; EKO Instruments Co., Ltd., Tokyo, Japan). DC power supplies (PAS60-4.5 for blue LEDs, and PMC35-1 for red and white LEDs; Kikusui Electronics Corp., Yokohama, Japan) were used to supply electrical currents to the LEDs, and the PPFD values of blue and red light were adjusted through current control. Digital timers (H5CX; OMRON Corp., Kyoto, Japan) were connected to the DC power supplies and used to remotely control the durations of blue and red irradiation.

## Results

### Experiment 1: Effects of Blue-Light Mono-Irradiation Duration

**Figure 4:**
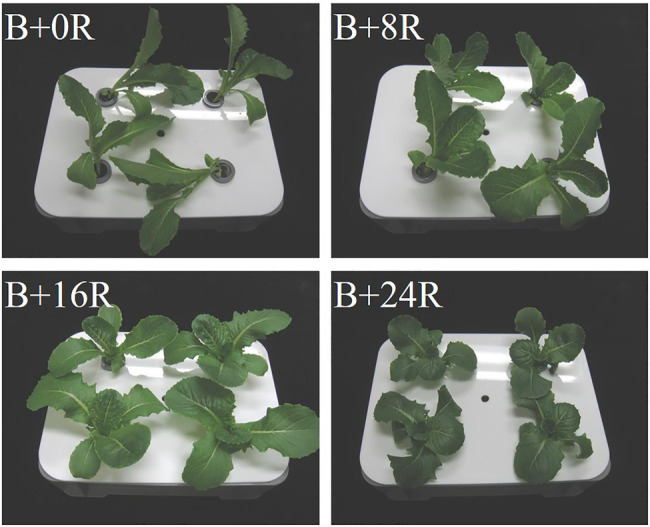
Cos lettuce plants grown under irradiation conditions described in Figure 1 (Experiment 1).

**Figure 5:**
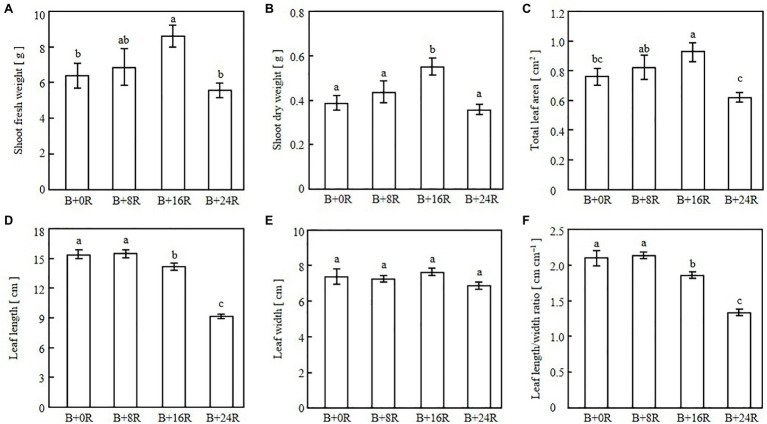
Shoot fresh weight **(A)**, shoot dry weight **(B)**, total leaf area **(C)**, leaf length **(D)**, leaf width **(E)**, and leaf length/width ratio **(F)** of cos lettuce seedlings grown under irradiation conditions described in Figure 1 (Experiment 1). Bars represent SEMs (*n*=8). Different lower-case letters above bars within a panel indicate a significant difference (*p*<0.05, Tukey–Kramer HSD test).

As the blue-light mono-irradiation duration increased, the leaves became more elongated (Figure 4). The average values of shoot fresh weight, shoot dry weight, and total leaf area were highest under B+16R and lowest under B+24R (Figures 5A–C). Leaf widths were similar among the treatments, but leaf lengths tended to increase along with blue-light mono-irradiation; thus, the leaf length/width ratio tended to be greater as the blue-light mono-irradiation duration increased (Figures 5D–F).

### Experiment 2: Effects of Blue-Light Irradiation Duration

**Figure 6:**
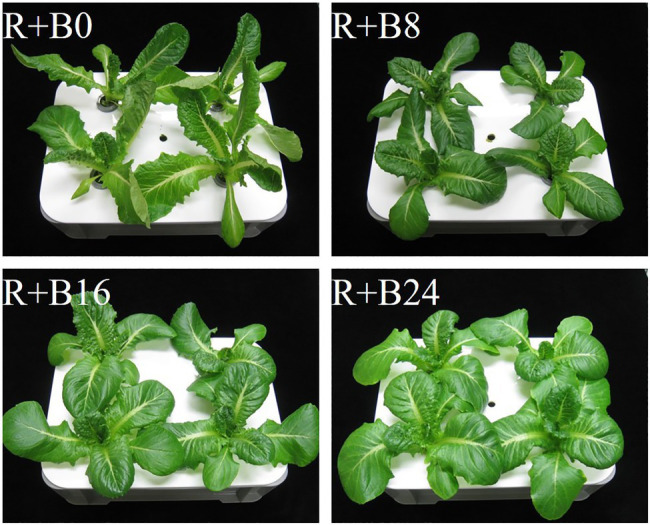
Cos lettuce plants grown under irradiation conditions described in Figure 2 (Experiment 2).

**Figure 7:**
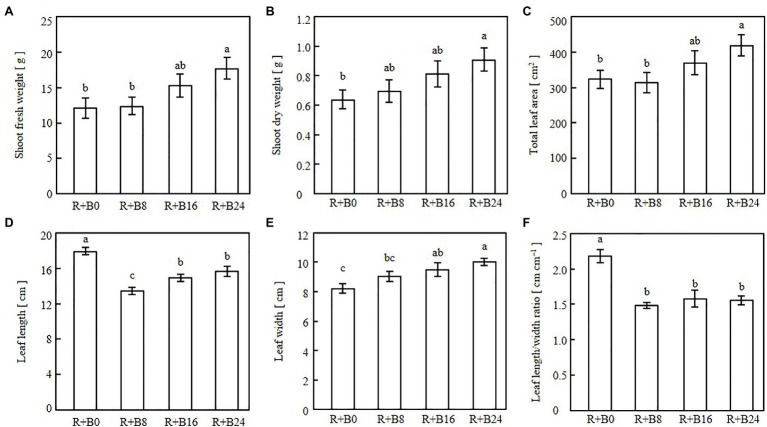
Shoot fresh weights **(A)**, shoot dry weights **(B)**, total leaf areas **(C)**, leaf lengths **(D)**, leaf widths **(E)**, and leaf length/width ratios **(F)** of cos lettuce seedlings under irradiation conditions described in Figure 2 (Experiment 2). Bars represent SEMs (*n*=8). Different lower-case letters above bars within a panel indicate a significant difference (*p*<0.05, Tukey–Kramer HSD test).

The leaves were elongated and twisted under R+B0 (Figure 6). In the blue-light irradiation treatments, the longer the blue-light irradiation, the flatter the appearance of the leaves, with no curling into bowl shapes. Shoot fresh weight, shoot dry weight, and total leaf area tended to be greater as the blue-light irradiation duration increased (Figures 7A–C). Leaf length was greatest under R+B0, and in the blue-light irradiation treatments; it increased along with the blue-light irradiation duration (Figure 7D). Leaf width tended to be greater as the blue-light irradiation duration increased (Figure 7E). As a result, the leaf length/width ratio was significantly greater under R+B0 and similar among the other treatments (Figure 7F).

## Discussion

### Experiment 1: Effects of Blue-Light Mono-Irradiation Duration

The leaf length may be increased under the longer blue-light mono-irradiation durations because of the resulting low PSS (Table 1; Sager et al., 1988), as shown by the elongation of cucumber stems (Hernández and Kubota, 2016) and lettuce leaves (Jishi et al., 2021) exposed to blue-light mono-irradiation. This result corroborates the finding by Jishi et al. (2016) that the longer the duration of both red-light mono-irradiation and blue-light mono-irradiation, the greater the promotion of cos lettuce leaf elongation. The present results clearly showed that leaf elongation was increasingly promoted by a longer duration of blue-light mono-irradiation. Thus, the degree of elongation mediated by the phytochrome reaction may be regulated by the duration of low PSS, such as blue-light mono-irradiation.

**Table 1:** Phytochrome photostationary state (PSS) values under several photosynthetic photon flux density (PPFD) combinations of blue- and red-LED light irradiation for each time slot of treatments in the present study.

Blue-/red-light PPFD (μmol m^−2^ s^−1^)	Treatment including the time slot with blue-/red-light PPFD	PSS
110/0, 100/0	B+0R, B+8R, B+16R, and B+24R	0.55
100/30	B+8R	0.86
100/15	B+16R	0.83
100/10	B+24R	0.80
30/100	R+8B	0.90
15/100	R+16B	0.90
10/100	R+24B	0.91
0/110, 0/100	R+0B, R+8B, R+16B, and R+24B	0.91

The PSS values were calculated from the spectral photon flux densities (Figure 3) and photochemical cross-section data of Sager et al. (1988).

The leaf widths were similar among the treatments, suggesting that the longer blue-light mono-irradiation duration resulted in elongated leaves rather than enlarged leaves. The leaf lengths under B+0R and B+8R were similar, but seedlings appeared spindlier under B+0R than under B+8R (Figure 4). The seedlings grown under B+0R collapsed owing to excessive elongation, and their dry matter production was low (Figure 5B) probably because of the low amount of light received. Therefore, leaf length under B+0R may have the potential to increase as a photomorphogenic response, but the biomass may have been insufficient to allow an increase in leaf length.

The differences in shoot fresh weight, shoot dry weight, and total leaf area may result from differences in the amount of light received. Under B+16R, the leaf surfaces facing the light source and the light-receptive area were high, whereas under B+0R and B+8R, the seedlings were tilted owing to excessive elongation. However, under B+24R, the leaves did not elongate notably and the light-receptive area was small. As a result, the degree of leaf elongation was moderate under B+16R for the purpose of promoting growth. Additionally, moderate durations of blue- and red-light irradiation vary depending on other environmental conditions. For example, the higher the DLI, the more plant elongation is suppressed (Johkan et al., 2012). If a similar experiment was conducted with a higher DLI, then elongation would be suppressed in all treatments and the leaf morphology of seedlings grown under B+8R or B+0R would be relatively “moderate.” The degree of plant elongation may be moderated by adjusting the duration of blue-light mono-irradiation in accordance with other environmental conditions, such as DLI.

### Experiment 2: Effects of Blue-Light Irradiation Duration

The significantly greater leaf length under R+B0 may result from the absence of the inhibitory effects of blue-light irradiation on elongation through the cryptochrome reaction (Ahmad et al., 1995; Zhao et al., 2007). The leaf length/width ratios, which were affected by blue-light mono-irradiation in Experiment 1, probably through the phytochrome reaction, were similar under R+8B, R+16B, and R+24B in Experiment 2. This suggests that the elongation-suppressive effect of the cryptochrome is affected by the presence or absence of blue-light irradiation during the day, but the effects of blue-light irradiation duration are small. The effects of blue-light DLI cannot be discussed for the present experiment because the blue-light DLI was identical among treatments. The blue-light DLI in this study (0.86mol m^−2^) may be sufficiently high to saturate the cryptochrome reaction. Wheeler et al. (1991) investigated the effects of blue-light PPFD in irradiated light on soybean grown under a 12-h light period. These authors found that stem elongation was suppressed as the blue-light PPFD increased up to 30μmol m^−2^ s^−1^ (1.30mol m^−2^), at which point the suppressive effect was saturated.

The differences in shoot fresh weight, shoot dry weight, and total leaf area resulted from differences in the amounts of light received. The treatments with a longer blue-light irradiation duration resulted in flatter leaves, which potentially have a greater light-receptive area by orienting the leaf surface toward the light source compared with that of epinastic leaves. Under R+B0, some leaves turned and did not face the light source, which likely also decreased the light-receptive area. Among the three treatments including blue-light irradiation, leaf length and width increased together with blue-light irradiation duration, whereas the leaf length/width ratio was similar. This may be because the development of flattened leaves was caused by the phototropin reaction, and the gain in biomass was caused by the increased light-receptive area of the flattened leaves.

The flattening of leaves with increase in blue-light irradiation duration may be a phototropin-mediated response (Inoue et al., 2008). Although the blue-light DLI was identical among treatments, the degree of leaf flatness was affected by the duration of blue-light irradiation; therefore, the phototropin reaction may be affected by the duration of blue-light irradiation rather than by blue-light DLI. Thus, the phototropin reaction induced by blue light may not follow the reciprocity law, whereas the phytochrome reaction is induced by end-of-day far-red light irradiation (Zou et al., 2021). However, 10μmol m^−2^ s^−1^, the lowest blue-light PPFD applied in the present study, may have been sufficient to saturate the phototropin reaction. Thus, it is possible that the phototropin reaction follows the reciprocity law at a significantly lower blue-light DLI and PPFD than applied in the present study.

### Lighting Patterns to Promote Growth

Adjusting the duration of blue-light mono-irradiation to moderately elongate leaves is one way to increase the amount of light received and promote growth. However, leaf elongation is effective in increasing light-receptive areas only when leaves do not shade each other. Normally, immediately after planting of a crop, there is ample space between seedlings; during this phase, leaf elongation effectively promotes growth until shading occurs. In comparison, under high plant densities in which shading occurs earlier than in the present study, differences in growth may not be observed as strikingly as in the present study.

Another method to regulate phytochrome-mediated elongation is far-red-light irradiation (Meng and Runkle, 2019). Far-red-light irradiation has the advantages that the PSS can be changed markedly by adjusting the far-red photon flux density and that it does not require timers to control the duration of blue- or red-light irradiation independently. When using far-red light irradiation, attention must be paid to the following: far-red light has a low photosynthetic efficiency; it is difficult to control the PSS because a small difference in the far-red PFD greatly affects the PSS; and the PSS differs between the upper and lower parts of the canopy owing to the greater penetration of far-red light compared with red light. On the basis of this study’s results, the degree of elongation is also likely to be regulated by adjusting the duration of the far-red-light irradiation during the light period. Blue-light mono-irradiation and far-red irradiation should be used separately or in combination, depending on the purpose.

Long-blue-light irradiation during the day promotes the development of flat leaves and growth. The blue-light DLI was 0.86mol m^−2^ in the present study, and leaf flattening may be further promoted by a greater blue-light DLI. However, because the emission wavelength bands of AlGaInP-based devices, which have relatively high luminous efficiency levels, are in the red region of 640–680nm (Jung et al., 2021) near the absorption peak of chlorophyll, it is better to increase the red-light DLI for the purpose of increasing the amount of photosynthesis per the amount of light received with equal energy consumption. The price of the light source (initial investment cost) also cannot be ignored as well as the electricity cost for lighting (running cost). Light sources with wavelengths that are in high demand for general home lighting are low-priced as a result of mass production. Even if the relative SPFD is not optimal for plant growth, the use of inexpensive light sources may maximize profits in commercial plant factories. The wavelength band of the light source should be selected with consideration of the estimated photosynthetic rate and photoreceptor reactions based on the absorption spectrum of the photoreceptors.

Photosynthesis is suppressed and growth slows if the light environment does not change during a close to 24-h cycle (Dodd et al., 2005). One reason that growth was suppressed under B+24R during Experiment 1 may have been because it was not a 24-h cycle. However, because growth was not particularly suppressed under R+B24 during Experiment 2, the effects of the circadian rhythms should have been small. Alternatively, cyclic changes in the PSS or the presence or absence of red light may be the main circadian rhythm-related signals.

We have discussed the photoreceptor reaction as the response of plants to a light environment comprising different SPFDs in different durations. However, detailed physiological experiments, such as those using mutants, are needed to test these hypotheses. In addition, growth analysis is useful to examine the effect of the light environment on growth rate through morphology. Conducting such studies in the future, and the possible manipulation of plant responses using a time-varying lighting method, are predicted to lead to further advances in light irradiation in plant factories.

## Conclusion

The leaf morphology and growth of cos lettuce were affected by the durations of blue- and red-light irradiation. Leaf elongation increased along with the blue-light mono-irradiation duration, and leaf flattening increased along with the blue-light irradiation duration. These morphological traits can be applied to increase the amount of light received, thereby promoting the growth of cos lettuce.

## Data Availability Statement

The raw data supporting the conclusions of this article will be made available by the authors, without undue reservation.

## Author Contributions

TJ conducted the experiments and wrote the manuscript. RM and KF supervised the experiments, provided editorial advice, and revised the manuscript. All authors contributed to the article and approved the submitted version.

## Conflict of Interest

The authors declare that the research was conducted in the absence of any commercial or financial relationships that could be construed as potential conflict of interest.

## Publisher’s Note

All claims expressed in this article are solely those of the authors and do not necessarily represent those of their affiliated organizations, or those of the publisher, the editors and the reviewers. Any product that may be evaluated in this article, or claim that may be made by its manufacturer, is not guaranteed or endorsed by the publisher.
